# Measuring the long-term effects of informal science education experiences: challenges and potential solutions

**DOI:** 10.1186/s43031-021-00031-0

**Published:** 2021-04-30

**Authors:** Nancy L. Staus, John H. Falk, Aaron Price, Robert H. Tai, Lynn D. Dierking

**Affiliations:** 1 STEM Research Center, Oregon State University, Corvallis, OR USA; 2 Institute for Learning Innovation, Portland, OR USA; 3 Museum of Science and Industry, Chicago, IL USA; 4 University of Virginia School of Education and Human Development, Charlottesville, VA USA; 5 College of Education, Oregon State University, Corvallis, OR USA

**Keywords:** Analytic approach, Attribution, Attrition, Informal science learning, Longitudinal, Methodology, Person-centered, Prospective, Retrospective, Variable-centered

## Abstract

Despite the fact that most science learning takes place outside of school, little is known about how engagement in informal science learning (ISL) experiences affects learners’ knowledge, skill development, interest, or identities over long periods of time. Although substantial ISL research has documented short-term outcomes such as the learning that takes place during a science center visit, research suggests that the genuine benefits of informal experiences are long-term transformations in learners as they pursue a “cascade” of experiences subsequent to the initial educational event. However, a number of major methodological challenges have limited longitudinal research projects investigating the long-term effects of ISL experiences. In this paper we identify and address four key issues surrounding the critical but challenging area of how to study and measure the long-term effects or impacts of ISL experiences: attribution, attrition, data collection, and analytic approaches. Our objective is to provide guidance to ISL researchers wishing to engage in long-term investigations of learner outcomes and to begin a dialogue about how best to address the numerous challenges involved in this work.

## Introduction

It is becoming increasingly clear that learning is a distributed process taking place across multiple settings, timescales, and activities (Bronfenbrenner, 1979; National Academies of Sciences, Engineering, and Medicine, 2018). Such is certainly the case for science learning, with substantial evidence indicating that people learn science cumulatively across a diversity of physical and social contexts including school, home, museums, libraries and science programs (National Research Council, 2009, 2010). However, given that most people spend only a small fraction of their life in school, out-of-school experiences such as visiting science centers or watching nature documentaries appear to be particularly important in contributing to people’s science understandings over time (Falk & Dierking, 2010; Sosniak, 2001). Collectively, the science learning that takes place outside of school is referred to as informal science learning (ISL) which includes a wide variety of experiences comprising three major categories: everyday environments (e.g., watching TV or reading the newspaper), designed environments (e.g., museums, science centers), and programs (e.g., science clubs, citizen science activities) (National Research Council, 2009). Because virtually everyone engages in ISL activities on a regular basis, from reading a news article about climate change or looking up health information on the internet to visiting a science museum or tending a garden, people accumulate science knowledge throughout their lifetimes, not just the years they are in school.

Accordingly, scholars have begun examining science learning from an ecological perspective, in which the educational “ecosystem” is conceptualized as a set of environments, both physical and virtual, in- and out-of-school, that provide people of all ages and backgrounds with opportunities to learn (Barron, 2010; Corin, Jones, Andre, Childers, & Stevens, 2017; Falk, Dierking, Staus, et al., 2016; Jackson, 2013; Traphagen & Traill, 2014). Within an educational ecosystem, learners construct unique learning pathways, guided by personal goals and interests and supported by myriad sociocultural and physical factors including activities, resources, and relationships that the learner can engage with to support their personal interests and learning goals (Barron, 2006; Bathgate, Shunn, & Correnti, 2014; Crowley, Barron, Knutson, & Martin, 2015; Jackson, 2013).

Despite the long-term, cumulative nature of informal science learning, most ISL research to date has been limited in both duration and scope, designed to document the results of short-term experiences such as the learning outcomes from a single science center visit or similar event, rather than documenting ISL pathways over time (National Research Council, 2015). While such research has provided valuable insights into what and how people learn during ISL experiences, it does not adequately capture the accumulation of knowledge and understandings derived from multiple sources over time both prior and subsequent to any particular educational event (Bransford, Brown, & Cocking, 2000; Falk, Koke, Price, & Pattison, 2018).

In order to advance the ISL field, longitudinal studies and innovations in assessment are essential to document the construction of STEM learning pathways in order to illuminate how STEM interests and understandings originate and evolve over time across a variety of ISL contexts and settings (National Research Council, 2015). However, these efforts have been significantly hampered by several methodological challenges related to the cumulative and personally unique nature of ISL learning that makes it difficult to study over significant periods of time. For example, unlike long-term studies in formal school-based environments where the curriculum is the same for all students, informal science learning takes place outside school during everyday activities and in designed spaces and programs such that learning pathways are unique to each individual, making it difficult to attribute learning outcomes to particular events or experiences (Falk, Koke, et al., 2018). In addition, while attendance is compulsory in formal spaces such as classrooms, ISL learners choose what, when and how long to participate in programs and other activities, making it difficult to follow individuals over time and increasing the chance of potential measurement issues due to attrition (Taplan, 2005).

The choice of appropriate data analysis approaches is also non-trivial. Historically, the most common research methods have involved a pre-post design using surveys and/or interviews administered immediately preceding and following an educational event to measure changes in knowledge, attitudes, and similar outcomes, presumably as a consequence of the experience. But these methodologies are unlikely to adequately capture the learning that takes place over significant time spans and across multiple settings and contexts. In addition, current methodologies may not adequately account for differences in outcomes that are attributable to learner heterogeneity because ISL learners are comprised of members of numerous different socio-cultural groups whose science explorations are shaped by their unique backgrounds and lived experiences reflecting a diversity of science perspectives, requiring researchers to utilize analytical approaches that account for this diversity (National Research Council, 2009). Clearly, researchers will need to develop innovative methods to assess science learning over longer time frames to address these challenges in future longitudinal ISL studies.

In this position paper, we address four key issues surrounding the critical but challenging area of how to study and measure the long-term effects or impacts of ISL experiences illustrated with examples from our own longitudinal ISL research efforts. While these discussions may be most useful for early-stage scholars who are less familiar with these issues, we believe they will also be instructive to more seasoned researchers who grapple with these issues in their own work. The first three sections discuss data collection challenges associated with long-term studies in general, but that are particularly difficult in ISL due to the personally unique and non-compulsory nature of informal learning experiences as described above. The final section examines how the choice of analytical approaches can influence interpretation of longitudinal data and associated outcomes. We summarize each section below:

In the first section, Falk discusses the issue of attribution within the context of long-term studies of ISL. Because learners engage in multiple ISL activities over time, it can be difficult to measure the relative contribution of any one experience to an individual’s knowledge, interest or behavior change over time. Falk provides an example of how attribution could be assessed from his work at the California Science Center.

In section two, Price and Tai address attrition, that is how longer time frames can lead to learners dropping out of studies which can potentially introduce selection, response, and other types of biases into the data. The authors provide an example of a long-term ISL study to illustrate why attrition may occur, and how they addressed the issue in their 8-year study.

In section three, Dierking discusses longitudinal data collection and the strengths and weaknesses of two methodologies commonly used in longitudinal studies: prospective, retrospective approaches.

In the final section, Staus describes how the choice of analytic models for measuring changes in outcomes over time are particularly salient due to the heterogeneous nature of participants in ISL programs and activities. She discusses the affordances and constraints of variable-centered and person-centered analytic models and provides an example of both types of analyses using an existing longitudinal data set.

## Attribution

When measuring the long-term effects of informal learning experiences, or any learning experiences for that matter, serious issues arise when trying to assign a causal relationship to outcomes. In other words, to what extent is it reasonable to assume that measured outcomes and effects for a specific informal learning experience can be directly attributed to participant engagement in that particular experience? Did these experiences actually “cause” observed outcomes or did they merely contribute to those outcomes?

It was once assumed that learning in general and science learning in particular was a fairly straightforward and linear process that primarily occurred through directed instruction – basically, the absorption-transmission model (cf., Bransford et al., 2000; Roschelle, 1995). However, learning is now understood to be neither straightforward nor linear. According to a recent OECD publication (Dumont, Istance, & Benavides, 2012), today the dominant view of learning is a socio-constructivist view, in which “learning is understood to be importantly shaped by the context in which it is situated and is actively constructed through social negotiation with others.” From this perspective, any particular learning experience, whether it takes place within a living room, classroom or a science museum, is almost certainly influenced by a host of other learning experiences occurring both previously and often subsequently. Thus, the ultimate outcome/effect of a particular learning event is likely to be only partially a consequence of that specific event. A full accounting of even short-term effects would require knowing something about each learner’s unique learning history prior to and after an event, and then only by viewing these trajectories in the aggregate could some understanding of the overall outcomes/effects of that event be inferred (cf., Falk, 2004).

As discussed earlier, learning is rarely instantaneous (National Academies of Sciences, Engineering, and Medicine, 2018). Individuals typically develop an understanding of and appreciation for science through an on-going and cumulative accumulation of experiences and understandings derived from multiple sources and situations (e.g., Anderson, Lucas, Ginns, & Dierking, 2000; Barron, 2006; Bathgate et al., 2014; Ito et al., 2013; National Research Council, 2009; 2015). For example, an individual’s understanding of the physics of flight might represent the cumulative experiences of completing a classroom assignment on Bernoulli’s principle, reading a book on the Wright brothers, visiting a science center exhibit on lift and drag, and watching a television program on birds. For the individual, all of these experiences are combined, often seamlessly, as they construct a personal understanding of flight; no one source is sufficient to create understanding, nor one single institution solely responsible. In the above scenario, when did this individual learn about flight, what experiences most contributed to learning? And how could one specifically identify and attribute the pieces learned while at the science center, for example, as opposed to the pieces learned in school, reading, or television? In other words, science learning is neither linear nor easily isolated in time and space.

Thus, it should not be unreasonable to assume that in most cases, measurable changes in outcomes are likely the consequence of contributory factors, rather than causative factors. This is not a trivial or merely semantic issue since arguably one of the primary goals of educational research is to allow educators and policy-makers to use findings as part of informed, evidence-based decision-making. Since research findings do indeed regularly figure into decisions about both the efficacy and cost-benefits of different types of educational experiences and practices, it would seem incumbent upon researchers to design studies in ways that allow for valid assessments of these relationships, whether directly causal or indirect, and hence correlational.

Reinforcing the importance of clarity around causation, a series of recent studies indicated that informal learning experiences significantly contributed to the public’s science learning (Falk, Dierking, Swanger, et al., 2016; Falk & Needham, 2011; Falk, Pattison, Meier, Bibas, & Livingston, 2018; Garg, Kauppi, Urajnik, & Lewko, 2007; Roth & van Eijck, 2010), and in some cases even appeared to be the major contributor to learning (Falk & Needham, 2013). Critics have countered by suggesting that these observed effects were primarily attributable to self-selection bias or methodological flaws (e.g., Jensen & Lister, 2016; Marino, Lilienfeld, Malamud, Nobis, & Broglio, 2010). These issues have been addressed elsewhere (Falk & Needham, 2016), but a brief discussion regarding self-selection is important because, of course self-selection is always a possible bias when studying informal experiences, since these types of experiences encourage user choice and control. Self-selection was specifically addressed in a large-scale, multi-national study of the effects of science center use (Falk, Dierking, Swanger, et al., 2016). Results convincingly showed that participation in these types of informal experiences significantly enhanced learning, independent of any confounding contribution of user’s prior interest, education and income. Clearly, adequately resolving questions such as these related to attribution are important, both for the educators working in these spaces (e.g., National Research Council, 2009; 2015), but also policy makers and funders, who historically have been skeptical about investing significantly in education initiatives outside the formal school system (Falk & Dierking, 2010).

Traditionally, researchers have approached the issue of causality and attribution by focusing on things like temporal order, measuring correlation, and accounting for confounding factors or alternative explanations (Campbell & Stanley, 1967; Fu, Kannan, Shavelson, Peterson, & Kurpius, 2016; Shadish, Cook, & Campbell, 2001). However as outlined in a recent whitepaper (Pattison, Dierking, Tai, & Kisiel, 2018), other perspectives on causality and approaches to studying causal relationships have emerged that recognize the complexity of learning in the real world (Gates & Dyson, 2017; Lemke, Lecusay, Cole, & Michalchik, 2015).

For example, some perspectives emphasize feedback loops and emergent properties within complex systems, rather than linear relationships, or highlight the challenges of self-report and reliability of participant narratives of causal chains (Falk, 2018). In the field of evaluation, there has been a growing focus on contribution rather than attribution, recognizing that the impact of any single program or initiative will be influenced by the variety of other experiences in a person’s life, before, during, and after the program takes place (Gates & Dyson, 2017). Research in informal learning settings has consistently highlighted the important influence of what individual participants bring with them to the experience and what happens to them afterwards (Falk & Dierking, 2019; Falk & Meier, 2018). Even within a traditional framework of thinking about causality, causal relationships almost always represent averages or probabilities, rather than universals, certainties, or inevitabilities. Modern statistical techniques allow researchers to model and test complex causal chains, multiple contributing factors, and mediating and moderating relationships, which often reveal the nuances underlying simple relationships between experience and outcome.

One relatively novel approach to the attribution issue has been the effort by Falk and his colleagues (Falk, Brooks, & Amin, 2001; Falk & Needham, 2011, 2016; Falk, Storksdieck, & Dierking, 2007) for over nearly two decades to directly measure the effects a specific science center – the California Science Center in Los Angeles, USA – has had on public understanding of science. In order to directly address the attribution issue, starting in the mid-1990s Falk identified what he called the learning equivalent of a radioactive tracer; “something that in and of itself may or may not be highly important, but which could be considered an indicator of something greater that was meaningful” (Falk & Needham, 2011, p. 3). Specifically, he and his colleagues focused on measuring the outcomes of a single experience at the science center; an experience that was designed to facilitate the learning of a single, relatively obscure concept, homeostasis. Research by Falk and Amin (1999) had shown that this particular experience effectively taught this single concept. Roughly three-quarters of all visitors sat through the 20-min presentation where Tess, a 50-ft animatronic woman and her animated sidekick Walt explained homeostasis. Whereas prior to the show, less than 10% of visitors could define homeostasis, after seeing the show, 85% of these same individuals could now provide an acceptable definition of this concept.

Although homeostasis is a concept that virtually all high school age youth learn in school, most, by adulthood, have long forgotten the term as well as what it means. When the California Science Center opened its doors to the public in 1998, Falk and Amin (1999) determined a baseline understanding of this concept within the greater Los Angeles adult population of 7%, i.e., only 7 out of 100 individuals recognized the term and were able to correctly define it. Two years later in 2000, knowledge of this concept by Los Angeles adults had increased marginally, but still significantly to 10%. The evidence suggested that the vast majority of individuals able to correctly define this concept in 2000 had visited the California Science Center at some point in the preceding 2 years (Falk et al., 2001). A decade after being open, again via a city-wide random survey, Falk et al. (2007) found that the percentage of adults able to define homeostasis had grown to 20% of adults; again with previous visits disproportionately represented amongst those able to provide a reasonable definition of the term. In the most recent citywide survey, conducted in 2015, 17 years after the science center opened, correct responses to the question about homeostasis had now reached 36% of Los Angeles adults. Considerable effort was made to identify alternative sources of information that could explain this change. Ultimately, this rise in ability to correctly define homeostasis could not be adequately explained other than as a consequence of visiting the science center. In fact, the rise in ability to define this term in Los Angeles has completely paralleled a second long-term trend, growth in the percent of Los Angeles adult residents having visited the California Science Center at least once, rising from 40% in 2009 to 67% in 2015. The 2015 data showed that, as before, there was significant statistical correlation between being able to define this term and having visited the science center (Falk, Pattison, et al., 2018). Again, the intent of this research was not to demonstrate that the learning of this one particular concept was, in itself, important, but rather to validate the accompanying self-report data from these surveys showing significant learning from this setting. In fact, this line of research revealed that although most science center visitors self-reported learning many things as a consequence of their visit, they regularly under-reported learning about homeostasis. In other words, self-report estimates of learning from these settings are likely under-estimates rather than over-estimates of actual learning (Falk & Needham, 2011).

In general, it is clear that this is an issue that remains challenging, though essential if science education researchers are ever to be able to validly define the effects of educational experiences and interventions. A recent study group investigating this issue (Pattison et al., 2018) recommended that researchers seek to study and communicate more nuanced hypotheses about causality that better reflected the situated and contingent nature of context, situations and relationships. They also recommended that researchers need to take responsibility for being more transparent about methods and their connection to evidence and claims, as well as ensuring that any and all claims are framed in ways that appropriately acknowledge the limitations inherent in any study. In particular, it is important that investigators make clear the possibility, if not the likelihood, that observed long-term outcomes associated with any particular educational experience likely connect with and/or were influenced by events occurring both prior and subsequent to the experience in question. The bottom line is that all researchers need to use caution in both their language and assertions around causality.

This more cautious and humble approach to causal relationships is likely to be challenging but clarifying the limitations of any individual investigation is critical for the field. Arguably, the whole issue of attribution outlined above is but one of many examples of the challenges inherent in investigating and understanding lifelong science learning in all its true complexity. The challenges of attribution also help to highlight why so many of the broad assumptions and “understandings” that underlie current science education policy so often turn out to be flawed, it not downright misguided – including the misplaced assumptions about the singular importance of formal educational experiences.

## Attrition

One of the principal methodological concerns of longitudinal studies, particularly those in which participants are not compelled to attend, is attrition or drop-out rate (Bauer, 2004). High attrition lowers sample size, which impacts statistical power, selection bias and overall generalizability. Here we discuss the issue of attrition in the context of “Developing YOUth!,” an 8-year longitudinal study of alumni of the adolescent development program at the Museum of Science and Industry, Chicago, USA. The study follows three annual cohorts of program graduates through their college experience (and hopefully beyond). Using a quasi-experimental, mixed-method design, the goal was to investigate the impact of the program on graduates’ college experience, STEM career interest and overall relationship with science. Data collection involved annual surveys of all participants (*N* = 228), interviews with about 20–30 participants and a deeper ethnographic relationship with about ten participants.

After the fourth wave of data collection, the study was averaging an annual loss of 6%, below an expected loss of ~ 20%, which was a rough estimate based on past experience with longer-term data collection and the level of interest we expected participants to have with the study. As a rule of thumb, response bias usually creeps into longitudinal data when the attrition rate exceeds 5% and becomes a threat to validity when it exceeds 20% (Schulz & Grimes, 2002). Here we describe our efforts to minimize attrition in this study and discuss what worked well, what didn’t, and provide guidance for other researchers embarking on longitudinal ISL studies in the future.

### Loss of interest

Two of the primary sources of attrition are loss of interest and loss of contact. Three ways to maintain study interest include establishing early buy-in into the research plan, consistent communication about study progress, and use of adequate incentives. First, following advice from Ludlow et al. (2011), we began by explaining the study to the population while they were active in the program. A year before recruitment, we provided presentations to participants and their families about the study’s goals, theoretical background and methodology, and we emphasized why a longitudinal study in particular was required to answer our research questions. The hope was to build an esprit de corps that would make the participants feel like they are part of a unique and special project. In the competition for their attention, we wanted the study to rise above the din of surveys we are all bombarded with as part of everyday life. We believe we achieved this: in all three annual cohorts > 90% of qualified participants joined the study.

It was also our intention to maintain interest in the study by writing frequent updates to a blog for participants to read. At the start of the project, we had a great deal of content since there was so much new information to introduce. However, as the years went by most of our content became restricted to updates on data collection, staffing changes, and some early results. In addition, we had to be careful about sharing too many details to decrease the risk of biasing our responses while data collection was still underway. Thus, our original goal of posting a blog entry every month or so for the entire project did not materialize, prompting us to find other ways to stay in touch.

In year three, we experimented with a longitudinal experience sampling study in which we took some questions from the annual survey (ex: interest in STEM majors, whether race/ethnicity had an impact on any of their STEM class experiences that year) and texted them to participants once a month over a year to examine changes on those items between our annual data collection points. Although we did not find much change over these short intervals, this strategy did enable us to keep participants involved with the project. That year we had our lowest attrition rate and did not lose a single person from the wave one treatment group. Now we augment our blog posts with a text message about once a year or so.

We cannot overstate the impact of proper incentives in mitigating attrition in long-term studies. We found that incentivizing was an essential tool for demonstrating appreciation and respect for participants’ time and efforts over our multi-year project. In our experience at the Museum over the last decade, we found that incentives do not generally alter participants’ demographic makeup, but they do increase recruitment efficiency. Considering the impact attrition has on longitudinal studies, efficiency is essential because having more participants on the front end builds in more statistical resilience later. Of course, incentives cost money so they must be budgeted into the up-front costs of the study. Although it may be tempting to skimp on the incentive budget, given the significant personnel and other costs of longitudinal studies we feel the benefits of incentives far outweigh the costs.

### Loss of contact

Losing contact with participants is less challenging now than in the past, thanks in large part to social media and semi-permanent cell phone numbers. We minimized the chance of losing contact with participants in several ways. First, when we registered study participants, we asked for multiple forms of contact (with permission to use each of them) – including social media accounts. Because our participants were transitioning from youth to adulthood, we also asked for their parents’ contact information. Our study began with a 2014 retrospective longitudinal study of prior program alumni going back 10 years (Price, Kares, Segovia, & Loyd, 2019). To maximize participation in data collection, we sent the surveys to the alumni’s parents’ address and scheduled their arrival for the week of Thanksgiving (and the reminder postcards the week after Christmas), since that would be the time the youth were most likely to be at home. This strategy was quite successful: we received responses from 30% of alumni, higher than typical for retrospective studies that go back about a decade.

In addition to collecting multiple forms of contact information, we also devised a plan for how and when to contact participants to help minimize attrition. For example, each year our first survey solicitation e-mail consisted of a relatively lengthy invitation including a reminder with all the details of the study. For non-responders, we followed up with a much shorter, more informal (e.g., joke or cartoon) e-mail that could be easily read on a cellphone. We scheduled the follow up message to arrive at a different time of day and a different day of the week than the original, to account for differing schedules. For those who still did not respond, we then sent one or two additional text messages. Finally, non-responders were contacted via their social media channels. Although we did not resort to contacting family, we reserved this as an option if attrition increased. We dropped participants upon request or when they did not respond to two consecutive waves of data collection.

Finally, some participants got married, changed personal identities or experienced other life events that can lead to changes in their names. In those cases, we were able to track most people through online searches, but it was time consuming because it had to be done manually. Having social media contact information for these participants was critical since many social media companies, such as Facebook, will also search by prior names. The COVID-19 pandemic has also had an unexpected impact on attrition. Since most of our surveys were administered online with a sizable direct incentive, we did not expect the pandemic-related shutdown to impact our data collection significantly. In fact, we thought it may help due to participants being less mobile and having more spare time. However, our annual attrition rate doubled in 2020 (our fifth year of data collection) to 13%. We believe this is due to participants being overwhelmed with surveys (and e-mail in general), and also that the study is understandably taking on a lower priority due to the uncertainty and overall turmoil in people’s lives.

### Missing data

Given that all longitudinal studies will experience some level of attrition over time, it is important to have a plan for addressing missing data in statistical analyses. The most common approach for dealing with missing data is to identify and examine the demographic characteristics of the subsample for which there is missing data. This includes potentially important factors such as gender and race/ethnic identity, as well as other factors known by the researchers to potentially introduce bias into the remaining sample. This analysis tests whether the missing data subsample falls into three different categories: “missing completely at random” (MCAR); “missing at random” (MAR); and “not missing at random” (NMAR) (Little & Rubin, 2002; Rubin, 1987).

Many widely used statistical packages include missing data analytical tools to produce these findings. If the missing data are found to be MCAR, then the researchers may perform a listwise deletion of the data and carry on with the analysis using the remaining sample of complete data. If the missing data are found to be MAR, then the researchers should consider methods to impute the missing data. These approaches include applying the expectation maximization algorithm as well as the implementation of multiple imputation (Pedhazur, 1997). Expectation maximization is popular given its relative ease of use. This approach uses the existing data to calculate imputed values for missing data in an approach similar to linear regression analysis. Clearly, this approach has some important drawbacks including a general biasing toward the mean for subsequent results. Multiple imputation is an approach that generates numerous parallel data sets with the missing values imputed using a combination of maximum likelihood calculations and estimated error randomization. It is not uncommon for this approach to generate hundreds of parallel data sets with imputed missing values that in turn are used to generate hundreds of parallel analytical models that are then averaged and outputted by the statistical package. Multiple imputation is gaining wider acceptance with many sophisticated statistical software packages including this option.

Another approach to the analysis of longitudinal surveys with missing data is the person-period analytical approach (Singer, Willett, & Willett, 2003). There are two formats for managing longitudinal data, person-level data format and person-period data format. Person-level format collects the data for each individual within a single line of values in the data set. This technique means that if a single variable for a single individual in any one of multiple waves of data is missing, then that individual’s observations might be potentially list-wise deleted from an analysis. Person-period format collects the data for each person at each wave of data collection. If data were missing for a single individual in the second wave of data collection, only the data for that specific individual in that specific wave would be considered to have missing data, thus retaining the data for this individual for the other waves of data collection where the data are complete.

### Qualitative data

Although we have focused our discussion on quantitative data collection and analysis, our study utilized a quasi-experimental, mixed-method design, including both annual snapshot interviews and ethnographic relationships that ran throughout the project. While not the subject of this discussion, we want to emphasize the importance of longitudinal qualitative data collection in fully understanding a participant’s experience in educational research (Calabrese Barton & Tan, 2018; Hermanowicz, 2013). In our study, participant attrition has been low due to the strong personal relationships the participants formed with the researchers. However, we have had issues with another potentially important source of attrition: staff. We originally hired post-doctoral researchers for qualitative data collection, which led to high levels of staff turnover as researchers left for permanent positions elsewhere. Although the participants were amenable to talking to new people, the disruption in these relationships negatively impacted the conversations and, perhaps more so, our analysis of the data. Our solution was to transition that role away from a postdoctoral position to one of a traditional staff member.

### Cooking the soup

In the above study, we showed how the use of multiple retention strategies, contact methods and robust statistical techniques together offered the best chance of reducing the impact of attrition (Laurie et al., 1999). Therefore, we argue that all of these strategies and techniques should be combined in a holistic manner, like ingredients in a soup. In a review of longitudinal study literature, Robinson, Dennison, Wayman, Pronovost, and Needham (2007) identified 12 strategic themes that led to successful retention. We designed our retention strategy to cover as many of the 12 themes as applicable. For example, one theme is “study identity,” which involves creating a standard aesthetic design and communication theme so participants can associate it with a brand. Another theme is “community involvement,” which calls for reaching the community in the early stages of the design/pilot process, which we did over the year before we began data collection. Given the flexible and ad hoc nature of many informal learning settings, this throw-everything-in-the-pot strategy is probably the best way to be prepared for the unexpected. These strategies are not mutually exclusive; they simply require time and resources. If we had to make just one recommendation, it would be to plan ahead and budget accordingly. Recognize that this will take time and resources and make the argument to stakeholders that these investments are necessary if you want truly valid results.

## Data collection

Research design choices influence the progression of a longitudinal research study, including issues of attribution and attrition discussed earlier, as well as what findings can be reported, and what claims can be made based on research results. In this section, we focus on two data collection methodologies typically used by researchers conducting longitudinal studies: retrospective and prospective methods. We discuss each design and its potential affordances and constraints in relation to long-term ISL studies.

### Prospective studies

Prospective studies (also called cohort studies) are those in which groups of individuals (cohorts) are selected and followed over a specific period of time, to determine the outcomes being studied. No specific period of time is required for a prospective study to be considered longitudinal, although most are at least a year long, oftentimes several (Tooth, Ware, Bain, Purdie, & Dobson, 2005). For example, the Harvard Study of Adult Development (Shah, Barsky, Vaillant, & Waldinger, 2014) and the 1970 British Cohort Study (Abbott et al., 2008) have been collecting data for decades to better understand developmental issues such as mental and physical health, and healthy aging. Similarly, the National Longitudinal Study of Adolescent to Adult Health (Add Health), 1994–2008 (Harris & Udry, 1994-2008) is a longitudinal study of a nationally representative sample of U.S. adolescents in grades 7 through 12 during the 1994–1995 school year designed to examine participants’ social, economic, psychological, and physical well-being, with contextual data on the family, neighborhood, community, school, friendships, peer groups, and romantic relationships.

Prospective longitudinal studies include repeated observations of the same individuals, allowing researchers to examine changes in outcomes over time. As such, prospective longitudinal studies are considered superior to cross-sectional studies, in which observations are made at a single point in time, include different individuals (a “cross-section”) of the population and result only in “snapshots” of impact (Vu, 2015). Prospective longitudinal studies also eliminate the risk of recall bias, although at times research participants are recalling “near” past events (Ho, Lieberman, Wang, & Samet, 2012). Unlike a one-off research project which can provide a snapshot of people’s lives at one point, longitudinal studies follow individuals through time collecting data at different points, more like a photo album. They tell a story of people’s lives at a moment in time, but also over time, showing how individuals or families have changed within a wider social context.

Prospective studies provide a context from which to investigate phenomena of interest both broadly and deeply. For example, in terms of the extremely long studies described above, it is not uncommon for there to be what are called “sweeps,” times in which all cohort members are recontacted and observed. In the case of the 1970 British Cohort Study there have been ten sweeps, the most recent one in summer 2020 at age 50. Such studies also can spawn sub-studies. In the case of the Synergies study described below (Falk, Staus, Dierking, et al., 2016), there have been sweeps as we followed the original cohort into high school (Shaby, et al., in review) and one sub-study (Wyld, 2015) in which one of the interventions, an afterschool web-based game design program, was studied over a 10-week period, investigating youth identity-building.

Despite the many advantages of prospective data collection methodologies described above, there are also several limitations that can hinder their use in long-term studies. In particular, prospective longitudinal studies require a great deal of time and can be quite expensive to conduct. Thus, these studies often have small samples, which make it difficult to generalize the results to a larger population. In addition, as discussed at length in the previous section, attrition can be a tremendous issue.

### Retrospective studies

Because of the challenges of long-term prospective studies described above, many researchers rely on retrospective data collection methodologies in which one collects data on events that have already occurred, asking participants to look back and reflect upon the experience and its impacts at the time, as well as its influence in the present (Bernard, Killworth, Kronenfeld, & Sailer, 1984). Retrospective studies are generally less expensive and take less time than prospective studies but also are more prone to measurement error (Mann, 2003). The advantage of the retrospective design is its small scale, usually short time for completion, and its applicability for particular research topics, which would require study of very large cohorts in prospective studies.

For example, a retrospective approach was taken in the author’s recent project, Cascading Influences: Long-term impacts of informal STEM programs for girls (McCreedy & Dierking, 2013, 2015), designed in part to explore the potential long-term impacts of informal STEM programs on girls’ long-term STEM pathways and career choices. By contacting women who had participated in such programs up to several decades earlier, the authors were able to retrospectively examine how and why some women were influenced by the STEM experiences they engaged in as youth. This methodology also took advantage of the fact that the “true” impact of an experience may not be understood by a person at the time of the experience, but only afterwards, through subsequent opportunities that reinforce and support it (Falk, Scott, Dierking, Rennie, & Cohen Jones, 2004). Thus, if retrospective findings support outcomes, this can be an indicator of potential learning and evidence for the cascading influence of these experiences.

Retrospective studies such as the one described above are often undertaken for research in the field of informal science learning, particularly in the area of museum learning research in which retrospective studies have focused on visitors’ long-term memories of exhibitions (Anderson, Storksdieck, & Spock, 2007). However, as compared to prospective studies, retrospective studies suffer from several drawbacks. Most significantly, attribution is a major concern, given that considerable time has passed so it may be difficult for participants to remember which specific experiences supported their science interest or learning. In addition, recall bias may negatively affect results when research participants are unable to recall past experiences accurately.

As in any study, the research design including data collection methodology should be informed by the research focus and questions underlying the study. However, design choices are even more significant when choosing to conduct a longitudinal study since such studies are time-consuming, require significant commitment and resources to be effective, and consequently, are more expensive than other types of research studies. Therefore, we suggest that researchers carefully consider the trade-offs when choosing data collection methods for longitudinal ISL projects.

## Analytic approaches

In addition to the above methodological concerns, ISL researchers must grapple with how to analyze the data once they have been collected. As described earlier, the majority of ISL studies thus far have examined the effects of short-term interventions such as museum visits, field trips or afterschool programs, often using univariate inferential statistics (e.g., t-tests) to examine changes in learning outcomes between pre- and post-visits (National Research Council, 2015). As the number and scope of longitudinal investigations in informal science research begin to expand, effective strategies for analyzing prospective data over time will be needed. Historically, science education researchers have used a variety of techniques to analyze longitudinal data such as repeated measures analysis of variance, regression analyses and more recently, growth curve modeling to examine changes in outcomes of interest (e.g., learning) over time. The goal of these analyses is to describe average trajectories of change in a single variable and to identify covariates that predict divergence from that pathway (Laursen & Hoff, 2006). Together, these and similar analytic techniques are known as “variable-centered” approaches because they describe associations among variables and as such, they are appropriate for addressing questions about relative contributions that predictor variables make to an outcome variable (Bergman & Wangby, 2014).

However, there are important limitations to variable-centered analytic models including the fact that they are based on the problematic assumption of homogeneity within a population regarding how predictors operate on outcomes (Laursen & Hoff, 2006). In addition, variable-centered methods produce group-level statistics like means and correlations that are not easily interpretable at the level of the individual and do not help us understand how and why individuals or groups of similar individuals differ in their development or learning outcomes over time (Bergman & Lundh, 2015).

### Person-centered analytic models

It is increasingly clear that groups of learners are not homogeneous in terms of attitude, interest, motivation or a number of other oft-reported outcomes related to learning in both formal and informal settings (Andersen & Chen, 2016; Chow, Eccles, & Salmela-Aro, 2012; Sheldrake, Mujtaba, & Reiss, 2019; Staus, Falk, Penuel, et al., 2020). Therefore, some researchers are beginning to embrace “person-centered” analytic models that are predicated on the assumption that populations of learners are heterogeneous, and therefore best studied by searching for patterns shared by subgroups within the larger sample (Block, 1971). While variable-centered approaches see people as the medium through which variables operate, person-centered models treat variables as properties of individuals and those variables may be grouped or clustered differently in different types of people (e.g., personality types). Therefore, the focus is on identifying distinct categories or groups of people who share certain attributes that may help us understand why their outcomes differ from those in other groups (Magnusson, 2003). Frequently, these attributes are psychological constructs that are important in the learning process (e.g., attitudes, motivation).

Person-centered models are often used to examine group or individual differences in patterns of development or responses to interventions over time (Denson & Ing, 2014). Standard statistical techniques include profile, class, and cluster analyses. For longitudinal studies, these classification analyses can be extended to investigate changes in class membership over time and identify predictors of transitions across classes (Lanza & Collins, 2008). These analytic models are suitable for addressing questions about group differences in patterns of development and associations among variables (Laursen & Hoff, 2006).

Importantly, person-centered approaches do not compete with or replace variable-centered analyses. In fact, it is recommended that longitudinal designs incorporate both approaches to examine different aspects of human development. For example, a researcher might utilize profile analysis to identify categories of individuals within a population and then use variable-centered analyses to examine predictors or correlates of group membership.

### The case for using person-centered analyses in ISL

Person-centered analyses such as cluster or latent profile analysis offer several methodological advantages as discussed above, but in order to use them effectively they must be applied in a theory-driven way (Howard & Hoffman, 2018). In other words, the selected variables that form the profiles must have a strong conceptual basis and have the potential to form distinct categories that are meaningful for analyzing outcomes (Spurk, Hirschia, Wang, Valeroc, & Kauffeld, 2020).

In terms of informal science learning, this appears to be the case. Substantial research from informal science institutions such as museums, science centers, zoos and aquariums, indicates that visitors arrive with a variety of typical configurations of interests, goals, and motivations that are strongly associated with learning and visit satisfaction outcomes (Falk, 2009; Packer, 2004; Packer & Ballantyne, 2002). For example, Moussouri (1997) identified six categories of visitor motivations reflecting the functions a museum is perceived to serve: place, education, life-cycle, social event, entertainment, and practical issues. The following year, Falk, Moussouri, and Coulson (1998) used this typology to investigate visitor learning outcomes across motivation categories at a natural history museum and found that both education and entertainment goals were associated with greater learning than other motivation categories.

Packer (2004) expanded on this work in a study of educational leisure experiences including museums and interpretive sites, by using a quantitative survey methodology in which she identified five categories of visitor goals which she validated with a factor analysis: (1) passive enjoyment; (2) learning and discovery; (3) personal self-fulfillment; (4) restoration; and (5) social contact. Learning and discovery goals correlated positively with learning and interest development; no other relationships between visitor goals and learning outcomes were significant. Thus, both studies suggested learning outcomes differed based on visitor goals or motivations, supporting the potential usefulness of person-centered analyses in informal science research.

Although similar studies in other informal science learning settings are rare, a recent evaluation of 27 afterschool STEM (science, technology, engineering and mathematics) programs in Oregon revealed significantly different affective outcomes (e.g., identity, belonging, resilience) for youth in two different motivation-related clusters (Staus, O’Connell, & Storksdieck, 2018). Specifically, youth who entered the program with low motivation for learning STEM showed significantly greater gains in affective outcomes than those who were already highly motivated to engage in STEM programs and activities. Taken together, these examples suggest that person-centered methodologies are likely appropriate for longitudinal investigations of learning-related outcomes in informal science settings.

### Illustrative example

Although used frequently in other fields such as educational psychology, sociology, and vocational behavior research, person-centered analyses are fairly uncommon in science education research (Denson & Ing, 2014; Spurk et al., 2020). To help clarify the methodologies described above, we provide an empirical example in which each type of approach is used on the same longitudinal data set from the authors’ prior research (Staus, Falk, et al., 2020) and the usefulness of the person-oriented approach and the variable-oriented approach are compared.

The Synergies Project was a five-year longitudinal study designed to better understand and support youth interest and persistence in STEM during adolescence, particularly for groups historically under-represented in STEM (Falk, Staus, et al., 2016). As part of the mixed-methods study, we administered an annual survey to measure interest in four STEM components, both in and out-of-school--earth/space science, life science, technology/engineering, and mathematics--so we could track changes in STEM interest over time (Staus, Lesseig, Lamb, Falk, & Dierking, 2020).

**Fig. 1:**
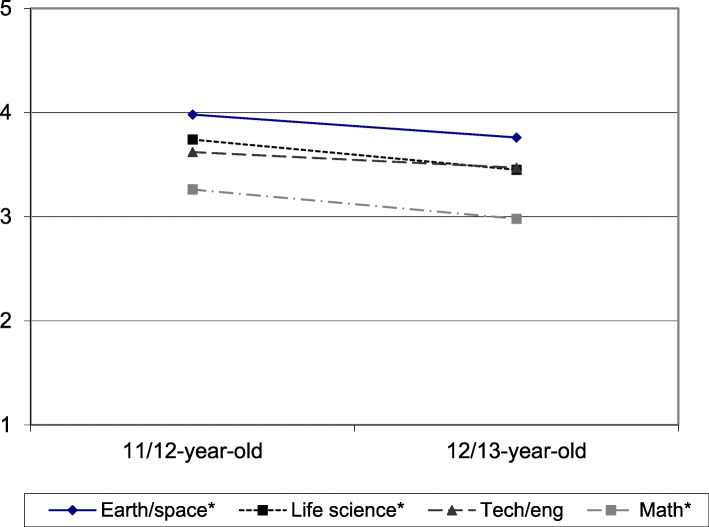
Changes in average interest in each STEM component for 106 youth aged 11/12 (sixth grade) to 12/13 (seventh grade). Interest scores ranged from 1 = “Not interested” to 5 = “Very interested.” Pathways with an asterisk denote significant differences at the *p* < 0.05 level

Previous researchers have used variable-centered techniques to examine mean changes in aspects of STEM interest over time and reported declining attitudes, motivation, or interest towards STEM topics and domains between ages 11–14, with declines most pronounced for girls (for reviews, see Osborne, Simon, & Collins, 2003; Potvin & Hasni, 2014). Figure 1 illustrates results of a similar variable-centered analysis of STEM interest data for youth in our study indicating that on average, youth lost interest in three of the four components of STEM between ages 11 and 13. A similar analysis based on gender showed that girls (*n* = 59) lost interest in earth/space science (t = 3.03; *p* = 0.004; d = 0.34) and mathematics (t = 2.11; *p* = 0.04; d = 0.31) whereas boys (*n* = 47) lost interest only in life science (t = 2.25; *p* = 0.03; d = 0.38) over this time period. Thus, our variable-centered analysis reinforced the prevailing notion about extensive youth interest declines during adolescence, particularly for girls.

**Fig. 2:**
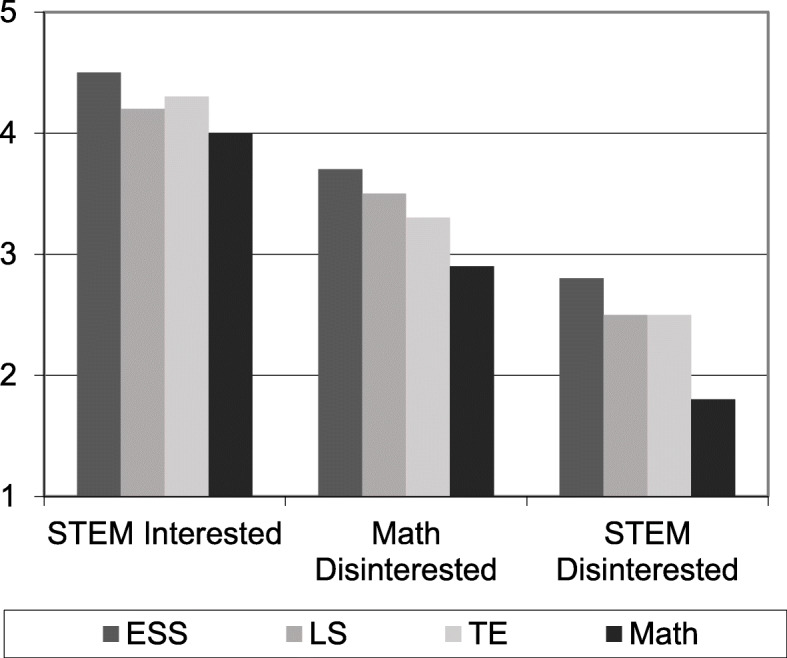
Interest scores for the four STEM components across the three STEM interest profiles for youth aged 12/13 (seventh grade). ESS = Earth/space science; LS = Life science; TE = Technology/engineering. Interest scores ranged from 1 = “Not interested” to 5 = “Very interested”

However, it was clear from our interview data that many youth did remain interested in STEM during this time period, but the variable-centered approach effectively erased their experience by treating the population as homogeneous in terms of STEM interest. Therefore, we conducted a cluster analysis using the four STEM interest components from the survey to identify unique STEM interest profiles for participating youth. This person-centered approach revealed three distinct profiles for youth aged 12/13-years-old: STEM Interested youth were characterized by significantly greater interest in all four STEM components than youth in the other profiles; Math Disinterested youth reported slightly positive to neutral interest in earth/space science, life science, and technology/engineering but slightly negative interest in math; and STEM Disinterested youth indicated the lowest interest in all four STEM components (Fig. 2). There were no gender differences within the STEM Interested and STEM Disinterested profiles, but girls were more likely than boys to identify with the Math Disinterested group (χ = 7.82, *p* = 0.020). In other words, the person-centered analysis revealed that there were as many STEM interested girls as boys, a finding that was not visible in the variable-centered approach.

**Fig. 3:**
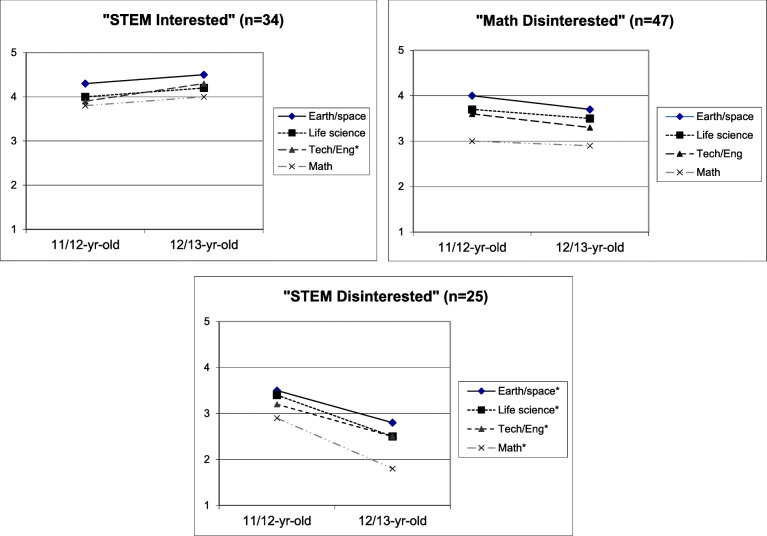
STEM interest pathways for youth aged 11/12 (sixth grade) and 12/13 (seventh grade) years-old by STEM Interest Profile. Interest scores ranged from 1 = “Not interested” to 5 = “Very interested.” Trajectories with an asterisk denote significant differences at the p < 0.05 level

We then examined how STEM interest changed between ages 11 to 13 for youth in the three interest profiles to see how they compared to average changes revealed in the variable-centered approach above. As depicted in Fig. 3, outcomes varied considerably for youth in the different profiles. Specifically, those in the STEM Interested profile reported a significant increase in interest in technology/engineering and maintained moderately high interest in the other three components. Youth in the Math Disinterested profile reported no significant changes in interest for any STEM components over time. Only youth in the STEM Disinterested profile showed a pattern of declining interest in all four STEM components. In other words, the person-centered approach revealed that for three quarters of youth, STEM interest remained the same or increased over time; the decline in STEM interest on average was being driven by the significant declines in STEM interest of the 24% of youth in the STEM Disinterested profile.

## Conclusions

The above example showed how variable-centered and person-centered approaches can provide results that lead to very different interpretations of the same data set. While the standard variable-centered analysis revealed an average developmental trajectory of declining STEM interest in the population, the person-centered approach allowed for different classes of subjects to follow different typical trajectories. While neither of the above analyses are “right” or “wrong,” in our opinion, the person-centered analysis provided information about longitudinal relationships at the pattern level that is potentially more useful in informing educational interventions that better support youth STEM interest development and persistence.

Ultimately, the choice of research strategies should be driven by the research question: variable-centered approaches can illuminate general principles that connect variables over time, whereas person-centered approaches are suitable for examining how life trajectories of some people differ from those of others, and a combination of both can provide complementary perspectives (Laursen & Hoff, 2006). However, considering the potentially great heterogeneity of informal science learners in terms of variables related to learning outcomes, it would behoove researchers to be aware of person-oriented approaches that may be more appropriate than standard variable-centered approaches for examining and understanding learning outcomes over time.

### Final words

Individuals learn science throughout their lives in a variety of contexts and settings. The cumulative nature of learning requires longitudinal studies to fully document the range of learning outcomes that emerge during the course of many science-related activities over long periods of time (National Research Council, 2015). While this presents challenges for investigating long-term learning outcomes in any context, it is particularly the case within informal spaces for a number of reasons outlined in this paper. In particular, the non-compulsory and personally unique nature of ISL experiences exacerbates issues of attribution and attrition. While the effects of attrition can be ameliorated somewhat with data collection techniques such as retrospective studies, these have their own disadvantages including potential inaccuracies in recall of past events. Finally, ISL learners are not homogeneous groups but are rather comprised of a variety of different socio-cultural groups whose learning outcomes may differ based on their unique backgrounds, requiring researchers to utilize analytical approaches that account for this diversity (National Research Council, 2009).

In this paper, we have provided brief overviews and discussions of each of these ISL research challenges illustrated with examples of longitudinal studies that have sought to address the issues of attribution, attrition, data collection and analytical approaches. We hope that our commentary will provide guidance to ISL researchers wishing to engage in long-term investigations of learner outcomes and will begin a dialogue among researchers about how best to address the numerous methodological challenges involved in this work.

## Data Availability

Not applicable.
